# How patients navigate specialist referral pathways in hand surgery: a cross-sectional survey study

**DOI:** 10.1186/s12913-026-14812-8

**Published:** 2026-05-30

**Authors:** Julius Asschenfeldt, Toni Engmann, Stephen A. Stearns, Michael F. McTague, Katiri T. Wagner, Arriyan S. Dowlatshahi

**Affiliations:** 1 Medical Faculty, Ruprecht-Karls-University, Heidelberg, Germany; 2 Division of Plastic Surgery, Department of Surgery, Beth Israel Deaconess Medical Center, Boston, MA USA; 3 Carl J. Shapiro Department of Orthopaedics, Beth Israel Deaconess Medical Center, 330 Brookline Ave, Stoneman 10, Boston, MA 02215 USA; 4 Harvard Medical School, Boston, MA USA

**Keywords:** Specialist referral, Patient decision-making, Primary care, Health services research, Healthcare access

## Abstract

**Background:**

Patients navigating specialist care are often presented with multiple options and information sources. In fragmented healthcare systems, understanding how individuals select specialists is important for optimizing referral pathways and access to care. This study examined factors influencing patient selection of hand surgeons, including the relative importance of primary care recommendations, surgeon characteristics, online information (such as physician rating websites and internet-based search resources), opinions of family and friends, as well as demographic differences in these preferences.

**Methods:**

This cross-sectional survey study was conducted among adults residing in New England. A 30-item online questionnaire was developed with input from hand surgeons and researchers and refined through pilot testing in lay participants. The survey was built in REDCap and distributed via ResearchMatch to 3,000 registrants, of whom 868 responded (response rate 28.9%). A total of 739 fully completed surveys were analyzed. Associations between demographic variables and factors influencing specialist selection were evaluated using chi-square tests, t-tests, and multivariable logistic regression analyses adjusting for demographic covariates. Statistical significance was defined as *p* < 0.05.

**Results:**

Primary care physician (PCP) recommendation (26%) and internet-based resources (24%) were the most frequently reported methods for identifying a hand surgeon. 83% of respondents reported that a PCP recommendation influenced their specialist selection. Increasing age was independently associated with decreased importance attributed to online physician ratings (*p* < 0.0001), opinions of family and friends (*p* < 0.001), and insurance-related factors (*p* < 0.0001). Most respondents reported a preference for orthopedic practice settings (77%) and orthopedic training backgrounds (90%).

**Conclusion:**

In this survey, PCP recommendations were the strongest reported influence on specialist selection, with age-related differences in the importance of online and interpersonal information sources. These findings suggest that primary care plays a major role in reported pathways to specialty hand care and that communication strategies may need to be tailored to different demographic groups within healthcare systems.

**Supplementary Information:**

The online version contains supplementary material available at 10.1186/s12913-026-14812-8.

## Background

Navigating specialist care within modern healthcare systems can be complex, particularly in regions where patients may access multiple provider types through both formal referral pathways and self-directed search strategies. Understanding how patients select surgical specialists is important for optimizing referral processes, improving access to care, and aligning healthcare delivery with patient preferences.

Hand surgery as a subspecialty originated less than 80 years ago as a collaboration between plastic and orthopedic surgery, with a substantial representation of orthopedic surgeons [1–3]. Given the varying training pathways to hand surgery and the wide variety of underlying hand pathologies, patients may have difficulty identifying the most appropriate specialist for treatment.

The decision a patient makes when selecting a physician can influence care pathways, referral efficiency, and healthcare utilization. Factors such as physician reputation, proximity to home or work, and insurance coverage influence decision-making [4, 5]. The growing availability of online resources has further expanded patient access to information about physicians and specialists [6]. However, how patients integrate traditional referral mechanisms, such as primary care recommendations, with digital information sources remains incompletely understood.

Patient expectations and physician perceptions may differ substantially [7]. A clearer understanding of how patients navigate specialist selection may help healthcare systems improve communication, referral alignment, and equitable access to specialty care.

This study explores factors influencing patient decision-making when selecting a hand surgeon and assesses public perceptions of various aspects of hand surgery. While existing literature primarily emphasizes surgical techniques, outcomes, and surgeon decision-making, patient perspectives on specialist referral pathways have received comparatively limited attention. The objective of this study was to identify the key factors influencing how patients select a hand surgeon, assess the resources used during this process, and examine demographic differences in these preferences.

## Methods

This cross-sectional survey study was conducted among adult residents of the New England region of the United States during early 2023 to evaluate factors influencing patient selection of hand surgeons. Ethical approval was obtained from the local Institutional Review Board (protocol #2021P000914).

A 30-question, web-based survey was developed to collect demographic information, including age, gender, race, education level, income, and health insurance status. Participants were asked which type of specialist they would approach for various hand conditions including pain, lacerations, fractures, infections, and amputations, and which clinical setting they would choose for initial evaluation. Additional items assessed how respondents would identify a hand specialist, which surgeon attributes they considered most important, and which factors influenced their selection.

The questionnaire was developed by the study team based on the study objectives and relevant literature on physician selection and referral pathways. To support content validity, the draft instrument was reviewed by a multidisciplinary group of researchers and board-certified hand surgeons. Formal psychometric validation of the instrument was not performed. The full questionnaire is provided in Supplementary File 1.

Several survey items used brief hypothetical clinical scenarios involving common hand conditions to assess respondents’ stated preferences for initial points of care and specialist selection. These scenarios were intended to capture anticipated decision-making rather than observed real-world behavior.

Study data were collected and managed using REDCap electronic data capture tools [8, 9]. REDCap (Research Electronic Data Capture) is a secure, web-based platform designed to support data capture for research studies, including validated data entry, audit trails, and export procedures compatible with common statistical software.

A survey link was distributed using ResearchMatch, an online research registry that allows investigators to contact volunteers who have agreed to receive study invitations. ResearchMatch emailed invitations containing a direct link to the survey to adult registrants residing in New England. Registrants were not selected based on prior hand pathology or prior hand-surgery consultation; invitations were sent to a broad convenience sample of ResearchMatch volunteers meeting geographic eligibility criteria.

Survey distribution was limited to adult residents of Connecticut, Maine, Massachusetts, New Hampshire, Rhode Island, and Vermont. Eligible participants were adults aged 18 years or older, residing in New England, and able to complete an online survey in English. No exclusions were applied based on prior hand pathology or surgical history. Participation was voluntary and anonymous, and participants were not compensated.

Primary outcome variables included patient-reported factors influencing hand surgeon selection, including primary care physician (PCP) recommendation, online physician ratings, surgeon location, insurance-related considerations, and surgeon training background. Predictor variables included age, gender, race, education level, income, and insurance type. All variables were obtained via self-reported responses entered directly by participants.

PCP recommendation was defined as agreement with the survey item assessing whether the opinion of the respondent’s regular doctor would affect surgeon choice. Online information was defined as internet-based resources used to identify or evaluate a hand surgeon, including general internet searches and online physician ratings. Insurance-related considerations referred to the reported importance of health insurance in surgeon selection, and surgeon location referred to the reported importance of geographic proximity. Opinions of family and friends were assessed as interpersonal influences on surgeon choice. Preferences regarding surgeon training background and practice setting were derived from items asking respondents where they would look for a hand surgeon and which training backgrounds they considered important.

To reduce targeted sampling bias, recruitment was not restricted by medical history or prior healthcare utilization. Participation was anonymous to minimize social desirability bias. Because ResearchMatch invitations were distributed anonymously, characteristics of non-responders could not be assessed; however, participation and completion numbers are reported to allow assessment of potential non-response bias.

No a priori sample size calculation was performed because the study was exploratory and the sample size was determined by the number of complete responses received during the distribution period. To provide a post hoc assessment of precision, the final analytic sample of 739 respondents provides an approximate 95% confidence interval margin of error of ± 3.6% points for a proportion of 50%, which represents the most conservative scenario for estimating survey proportions. Subgroup and regression analyses were considered exploratory.

Data were analyzed using R (Version 4.2.1). Quantitative variables were summarized as mean ± standard deviation, and categorical variables as frequencies and percentages. Age was analyzed using predefined survey categories (18–29, 30–39, 40–49, 50–59, 60–69, 70–79 and ≥ 80 years), selected a priori as clinically interpretable strata. Group comparisons were performed using Fisher’s exact tests, chi-square tests, and t-tests, as appropriate. Multivariable logistic regression models were constructed to evaluate associations between demographic variables and factors influencing specialist selection while adjusting for income, education, race, and gender. Survey responses assessing importance were dichotomized (agree vs. not agree) for regression analyses. Adjusted odds ratios with 95% confidence intervals were calculated, and statistical significance was defined as *p* < 0.05.

Only fully completed surveys were included in the primary analysis. Incomplete surveys were excluded. For regression analyses, complete-case analysis was performed for each outcome, resulting in variable sample sizes across models. No weighting, imputation, or formal sensitivity analyses were performed.

An exploratory post hoc subgroup analysis compared responses to the hypothetical clinical scenario items between respondents with and without a prior history of hand-related pathology and was considered hypothesis-generating.

This study is reported in accordance with the STROBE guidelines. A completed STROBE checklist is provided as supplementary material.

## Results

A total of 3,000 individuals were invited to participate through ResearchMatch. Of these, 868 individuals responded to the survey (overall response rate 28.9%). After exclusion of 129 incomplete surveys, 739 fully completed responses (24.6% of those invited) were included in the final analysis.

Participants represented all predefined age categories, with the 60-69-year age group most frequently represented (23%). 71% of respondents were female and 25% were male. Most respondents identified as White (83%), followed by 8.4% Black and 3.7% multiracial. 83% reported a college degree or higher. Household income ranged from less than $25,000 to greater than $150,000 (Table 1).

**Table 1:** Demographics of survey respondents

Total number of respondents	739
Age (%)	
18–29	102 (14)
30–39	152 (21)
40–49	95 (13)
50–59	102 (14)
60–69	169 (23)
70–79	102 (14)
80+	17 (2.3)
Gender (%)	
Female	528 (71)
Male	185 (25)
Other	26 (3.5)
Race (%)	
White	617 (83)
Black	62 (8.4)
Asian	12 (1.6)
More than one race	27 (3.7)
Other (Native/Pacific Islander/Other)	21 (2.8)
Ethnicity (%)	
Hispanic	39 (5.3)
Non-Hispanic	647 (88)
Other	53 (7.2)
Education (%)	
High school degree or less	33 (4.5)
Some college	101 (14)
College degree	270 (37)
Some graduate work, no degree	71 (9.6)
Graduate degree	264 (36)
Income (%)	
Less than $25,000	89 (12)
$25,000 - $49,999	126 (17)
$50,000 - $74,999	118 (16)
$75,000 - $99,999	102 (14)
$100,000 - $149,999	141 (19)
$150,000 or greater	163 (22)

Regarding healthcare characteristics, 96% of respondents reported active health insurance coverage, most commonly private insurance (51%). 91% reported having an established PCP. 60% had previously sought care for a hand-related issue, of which 72% were described as non-urgent (Table 2).

**Table 2:** Healthcare access and utilization characteristics

Insurance (%)	712 (96)
Type of Insurance (%)	
Public	174 (24)
Private	379 (51)
Workers Comp	11 (1.5)
Other	43 (5.8)
Multiple	105 (14)
PCP	671 (91)
Seen doctor for hand issue (%)	445 (60)
Emergency	89 (20)
Non-Urgent	321 (72)
Both	35 (7.9)

**Fig. 1:**
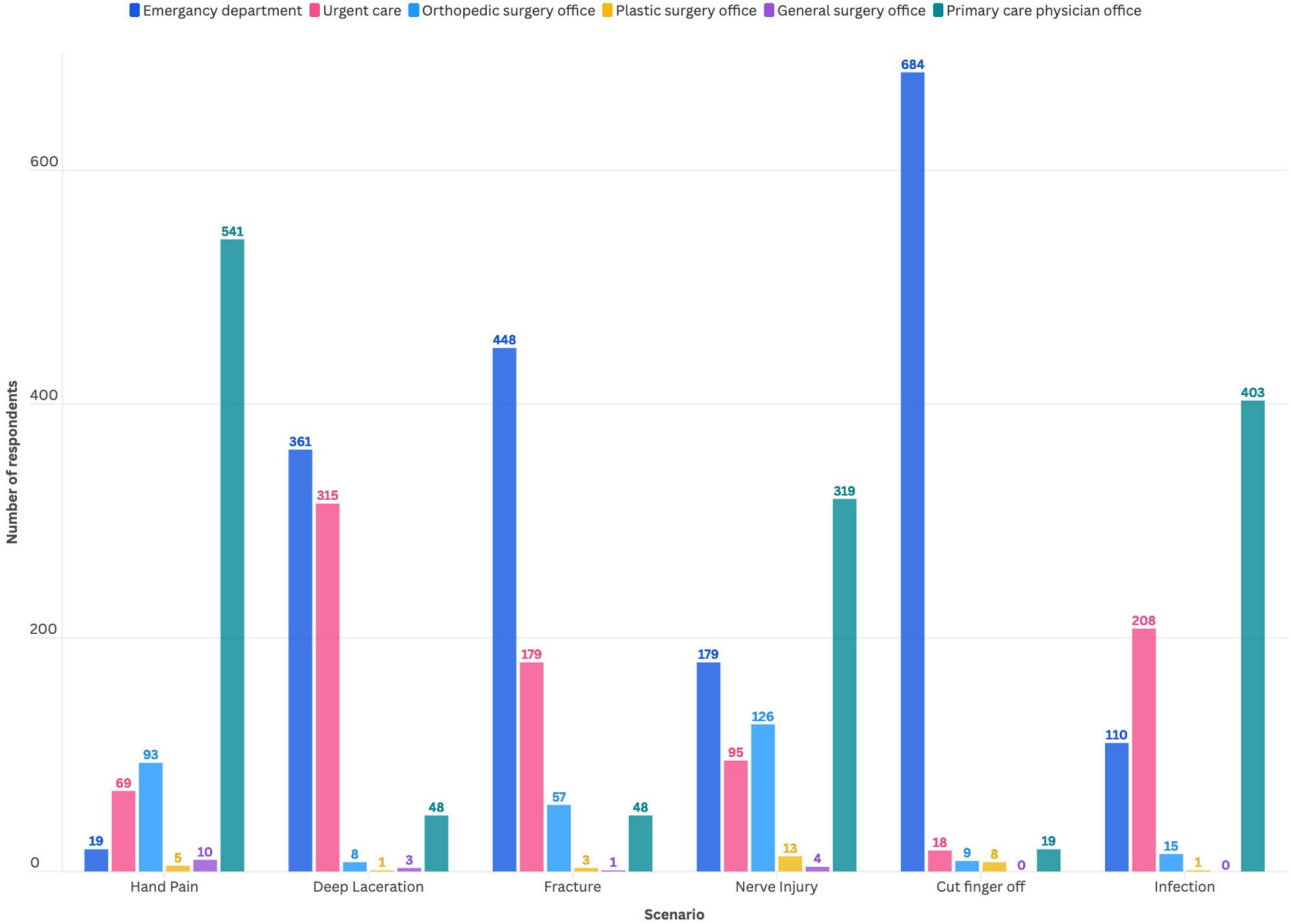
Initial resource used for varying hand pathology scenarios

Respondents were presented with multiple hypothetical hand pathology scenarios (Table 3). For finger amputations and deep lacerations, nearly all participants indicated they would seek initial treatment at an emergency department or urgent care. In contrast, for nerve injury, infection, and general hand pain, most respondents reported they would first present to their PCP. These patterns are further illustrated in Fig. 1.

**Table 3:** Hand scenario responses

First call – hand pain (%)	
Emergency department	19 (2.6)
Urgent care	69 (9.3)
Orthopedic surgery office	93 (13)
Plastic surgery office	5 (0.6)
General surgery office	10 (1.4)
PCP office	541 (73)
First call – deep laceration (%)	
Emergency department	361 (49)
Urgent care	315 (43)
Orthopedic surgery office	8 (1.1)
Plastic surgery office	1 (0.1)
General surgery office	3 (0.4)
PCP office	48 (6.5)
First call – fracture (%)	
Emergency department	448 (61)
Urgent care	179 (24)
Orthopedic surgery office	57 (7.7)
Plastic surgery office	3 (0.4)
General surgery office	1 (0.1)
PCP office	48 (6.5)
First call – nerve injury (%)	
Emergency department	179 (24)
Urgent care	95 (13)
Orthopedic surgery office	126 (17)
Plastic surgery office	13 (1.8)
General surgery office	4 (0.5)
PCP office	319 (43)
First call – cut finger off (%)	
Emergency department	684 (93)
Urgent care	18 (2.4)
Orthopedic surgery office	9 (1.2)
Plastic surgery office	8 (1.1)
General surgery office	0 (0)
PCP office	19 (2.6)
First call – infection (%)	
Emergency department	110 (15)
Urgent care	208 (28)
Orthopedic surgery office	15 (2.0)
Plastic surgery office	1 (0.1)
General surgery office	0 (0)
PCP office	403 (55)

In an exploratory subgroup analysis, responses to some hypothetical clinical scenarios differed between respondents with and without a prior history of hand-related pathology (Supplementary File 2). In particular, respondents with prior hand-related experience more often selected primary care or orthopedic surgery settings for some non-acute scenarios, whereas respondents without prior history selected urgent care or emergency settings more often.

The most frequently endorsed resources for identifying a hand surgeon were PCP recommendations (26%) and internet searches (24%) (Table 4). 83% of respondents reported that a PCP recommendation would affect their specialist selection. Physician online ratings and surgeon location were also commonly endorsed factors (Table 4).

**Table 4:** Resources used to identify a hand surgeon and preferences in surgeon selection

Which resources would I use to find a hand surgeon?	*n* = 739
PCP	192 (26)
Internet	177 (24)
Insurance	111 (15)
Hospital website	89 (12)
Call hospital	30 (4)
Ask friend	140 (19)
PCP referral to hand surgeon preferred	*n* = 710
Strongly disagree	25 (3.5)
Disagree	61 (8.6)
Neutral	124 (17)
Agree	209 (29)
Strongly agree	291 (41)
Prefer to find hand surgeon myself	*n* = 702
Strongly disagree	136 (19)
Disagree	163 (23)
Neutral	99 (14)
Agree	176 (25)
Strongly agree	128 (18)
Opinion of friends would affect choice	*n* = 699
Strongly disagree	81 (12)
Disagree	82 (12)
Neutral	132 (19)
Agree	319 (46)
Strongly agree	85 (12)
Opinion of regular doc would affect choice	*n* = 710
Strongly disagree	11 (1.5)
Disagree	25 (3.5)
Neutral	87 (12)
Agree	333 (47)
Strongly agree	254 (36)
Online surgeon ratings would affect choice	*n* = 693
Strongly disagree	48 (6.9)
Disagree	76 (11)
Neutral	171 (25)
Agree	310 (45)
Strongly agree	88 (13)
Health insurance would affect choice	*n* = 705
Strongly disagree	74 (10)
Disagree	81 (11)
Neutral	108 (15)
Agree	211 (30)
Strongly agree	231 (33)
Location of surgeon would affect choice	*n* = 698
Strongly disagree	34 (4.9)
Disagree	53 (7.6)
Neutral	156 (22)
Agree	301 (43)
Strongly agree	154 (22)

**Fig. 2:**
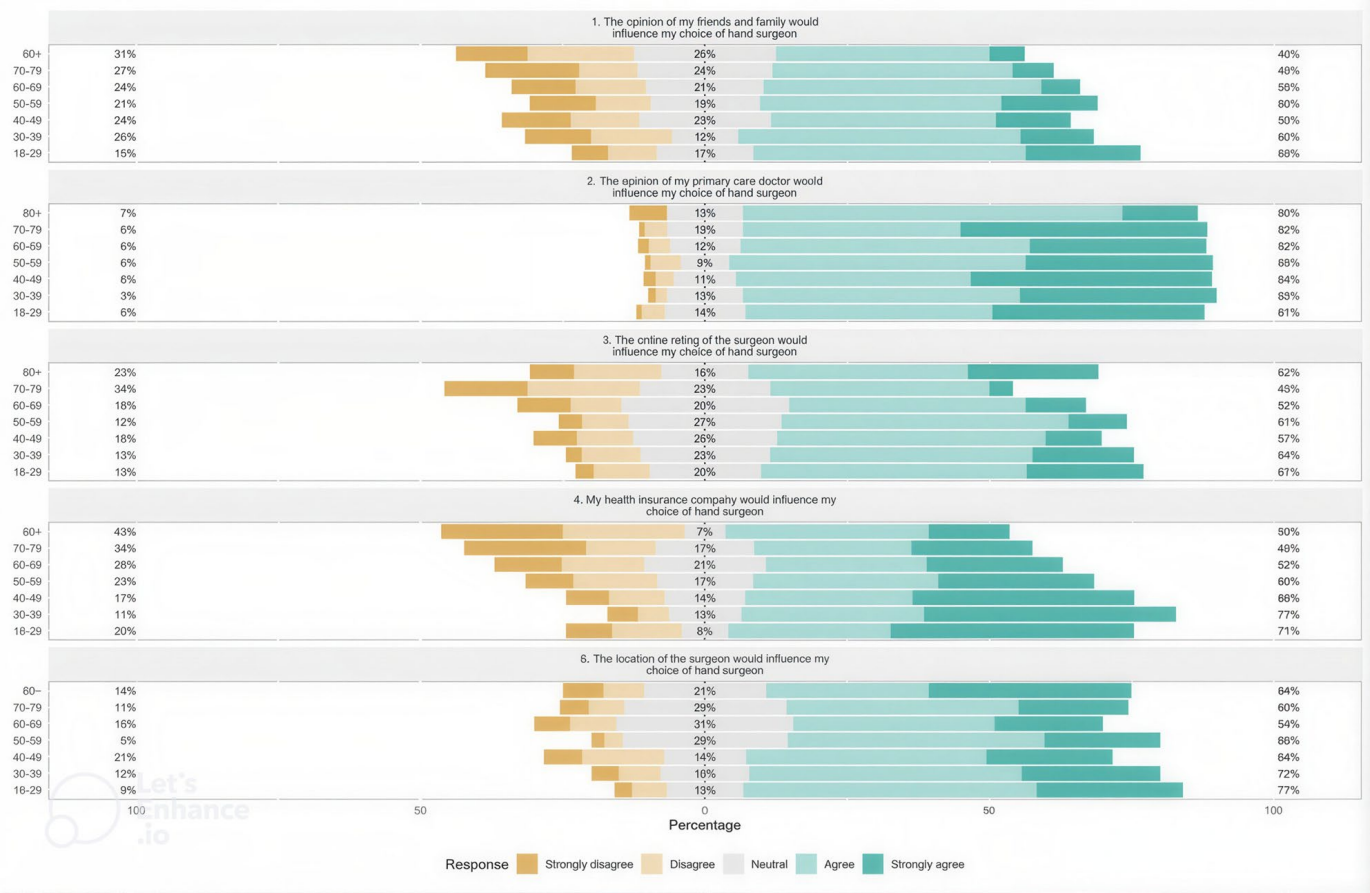
The effect of age on factors impacting hand surgeon selection

In unadjusted analyses, increasing age was associated with decreased importance attributed to online physician ratings (*p* < 0.0001), opinions of family and friends (*p* < 0.001), and insurance-related factors (*p* < 0.0001). These associations remained significant after adjustment for income, education, race, and gender. In multivariable logistic regression models, increasing age was associated with lower odds of attributing importance to online physician ratings (adjusted odds ratio [aOR] 0.85 per age category increase i.e. increase by 10 years, 95% CI 0.77–0.94; *n* = 687), opinions of family and friends (aOR 0.84, 95% CI 0.76–0.92; *n* = 692), and insurance-related factors (aOR 0.78, 95% CI 0.71–0.86; *n* = 699) (Fig. 2).

Insurance-related factors were significantly less influential among male respondents compared with female respondents (aOR 0.54, 95% CI 0.37–0.79; *p* = 0.0016).

No other independent associations were observed between the examined demographic variables and PCP recommendation, opinions of family and friends, or surgeon location.

Regarding practice setting, respondents were more likely to search for a hand specialist in an orthopedic surgery office (77%) than in a plastic/reconstructive surgery office (28%) (Table 5). In terms of training preference, 90% preferred orthopedic surgery training, 58% preferred microsurgical experience, and 54% preferred plastic/reconstructive surgery training (Table 5).

**Table 5:** Surgical training preference (multiple selections were permitted)

Look for a hand surgeon in a:	
Orthopedic surgery office	566 (77)
Plastic/reconstructive surgery office	204 (28)
General surgery office	102 (14)
Hand surgeons should have training in:	
Orthopedic surgery	662 (90)
Plastic/reconstructive surgery	396 (54)
Microsurgery	431 (58)
General surgery	345 (47)

## Discussion

Hand surgery encompasses a broad range of clinical presentations and training pathways, which may complicate patient navigation of specialist care. In this cross-sectional survey of adults in New England, we examined how respondents report approaching common hand conditions and how they identify and select a hand surgeon. For acute conditions such as finger amputations and deep lacerations, most respondents reported seeking initial care in emergency or urgent care settings. In contrast, for less urgent conditions such as nerve injury, infection, or general hand pain, respondents more frequently indicated they would first present to their PCP. These findings suggest that respondents differentiate between acute and non-acute hand conditions and report relying on different healthcare pathways for initial evaluation.

Exploratory subgroup analyses showed that respondents with prior hand-related pathology differed from those without prior experience in responses to some hypothetical scenarios. This suggests that prior contact with the healthcare system may influence stated care-seeking preferences and reinforces the limitation of using a general population sample rather than patients actively seeking care. However, since this analysis was not prespecified, it should be interpreted cautiously and viewed as hypothesis-generating rather than conclusive.

Although both orthopedic and plastic surgeons are trained to manage hand pathologies, respondents more frequently expressed a general preference for orthopedic practice settings and orthopedic training backgrounds. Prior work has demonstrated that orthopedic and plastic surgery training pathways differ in their emphasis on bony versus soft tissue reconstruction [10, 11]. These distinctions have contributed to the development of integrated orthoplastic service models that combine complementary expertise [12]. The preferences observed in this study may reflect general public familiarity with orthopedic surgery in the context of musculoskeletal injury rather than a detailed understanding of hand surgery subspecialty certification. Because survey questions were based on brief hypothetical scenarios, responses likely reflect initial perceptions rather than informed specialist comparisons.

A central finding of this study is the prominent role of PCPs in facilitating access to hand surgical care. PCP recommendation was the most frequently cited resource for identifying a hand surgeon and influenced specialist selection for most respondents. In addition, for several non-acute hand conditions, respondents reported they would first seek care from their PCP. These findings highlight the importance of primary care referral networks in shaping access to specialty care and suggest that strengthening communication and referral pathways between hand surgeons and primary care providers may improve care coordination.

Although 62% of respondents indicated that geographic location was an important factor, location was not independently associated with demographic variables in adjusted analyses. In this sample, the reported importance of geographic proximity appeared to be broadly distributed across respondent groups. However, because this study assessed stated preferences rather than observed behavior, it does not establish how strongly location influences actual specialist selection relative to other considerations. Prior studies examining physician selection have shown that travel distance may interact with perceived quality and referral patterns [13]. 

Online physician rating platforms represent an increasingly visible component of healthcare decision-making. However, prior research has questioned whether online ratings correlate with objective measures of quality [6, 14]. National survey data suggest that only a minority of patients actively use rating websites when selecting physicians [15]. In orthodontic care, personal recommendations have been shown to outweigh online reviews [16]. In contrast, our study found that online ratings and recommendations from friends or family were rated similarly in importance overall. Notably, increasing age was associated with lower odds of attributing importance to online ratings. These findings align with prior work demonstrating age-related differences in digital health engagement and online service utilization [17, 18]. 

Older respondents in our study were also less likely to prioritize recommendations from friends or family. This observation may reflect differences in social network structure or reliance on external information sources across the life course [19]. Additionally, prior behavioral research suggests that older adults may rely more heavily on personal experience and established healthcare relationships when making medical decisions [20]. These mechanisms may partially explain the reduced influence of external factors such as online ratings and insurance considerations observed in older age groups.

Importantly, the demographic composition of our sample likely underrepresents individuals with limited insurance coverage, lower educational attainment, or reduced access to primary care. Prior national data indicate that insurance coverage and socioeconomic status influence healthcare access patterns [21]. As such, the referral and decision-making pathways described here may not fully capture the experiences of disadvantaged populations. Future research should specifically examine how patients with limited access to primary care or digital resources identify and access hand surgical services.

Although this study was conducted in New England, its findings are relevant to broader health services questions faced across healthcare systems. Patients in many countries navigate between primary care referrals, specialist availability, self-directed information seeking, and online physician information when accessing specialty care. The relative importance of these pathways likely differs by healthcare system, including the degree of primary care gatekeeping, insurance structure, specialist availability, and digital health infrastructure. Therefore, while the specific proportions observed in this study should not be assumed to generalize internationally, the broader finding that specialist selection is shaped by both formal referral pathways and patient-directed information sources may inform future research and care-navigation strategies in other settings.

### Limitations

Several limitations warrant consideration. First, 96% of respondents reported active insurance coverage, which exceeds national estimates and may bias findings toward populations with stable healthcare access [21]. Second, a significant component of the survey relied on responses to hypothetical scenarios rather than observed behavior. Consequently, stated preferences may not accurately reflect real-world decisions made during acute or ongoing hand conditions, and the findings should be interpreted as perceptions of likely behavior rather than direct evidence of healthcare utilization. Third, participation through ResearchMatch may introduce selection bias toward individuals who are more engaged with healthcare, research participation, or digital resources. The response rate of 28.9% is within the range reported for online survey studies using volunteer recruitment platforms such as ResearchMatch [22], but it nevertheless introduces the possibility of non-response bias. Because the sample size was not determined by an a priori power calculation, null findings, subgroup analyses, and regression estimates should be interpreted cautiously. Fourth, the sampled population was not limited to patients actively experiencing or seeking care for a hand condition, which may further limit the applicability of these findings to real-world clinical decision-making in hand surgery. Finally, because this study was limited to residents of New England, generalizability to other regions with different referral structures or specialist availability may be limited. Although the questionnaire underwent expert review and pilot testing for clarity, formal psychometric validation was not performed, and some key constructs were assessed using single survey items. In addition, because preferences were elicited using brief hypothetical scenarios, question framing may have influenced some responses. Together, these factors may limit the reliability, reproducibility, and generalizability of the findings.

## Conclusion

Respondents in this regional survey most frequently identified PCP recommendations as a major influence when selecting a hand surgeon, supporting the importance of primary care in pathways to specialty care. Online ratings and informal recommendations were also commonly considered, but their importance varied by age. Respondents also more frequently expressed a preference for orthopedic practice settings and training backgrounds, while still valuing additional subspecialty expertise.

These findings support the importance of strong referral relationships between primary care and specialty services, as well as age-tailored communication strategies. Efforts to improve transparency, digital accessibility, and referral coordination may enhance patient navigation within musculoskeletal specialty care. Future studies should evaluate patients actively seeking care for hand conditions to determine how closely stated preferences align with actual referral and care-seeking patterns, as well as how underserved and underinsured populations identify and access specialty services.

## Supplementary Information

Below is the link to the electronic supplementary material.

Supplementary Material 1

Supplementary Material 2

## Data Availability

De-identified data that support the findings of this study are available from the corresponding author upon reasonable request and with appropriate ethical approval.

## Abbreviations

PCP: Primary care physician
aOR: Adjusted odds ratio
