# Supplementary material for: How patients navigate specialist referral pathways in hand surgery: a cross-sectional survey study
Source: BMC Health Serv Res. 2026 May 30;26:1058. doi: 10.1186/s12913-026-14812-8 (PMC13439914; doi:10.1186/s12913-026-14812-8)
Supplement: Supplementary file 1 [file 12913_2026_14812_MOESM1_ESM.docx]

| **Scenario** | **Response option** | **No prior hand pathology, n (%)** | **Prior hand pathology, n (%)** | **P-value** |
| --- | --- | --- | --- | --- |
| Fracture | Emergency room | 183 (65.8) | 255 (57.4) | 0.108 |
|  | Urgent care | 64 (23.0) | 112 (25.2) |  |
|  | Orthopedic surgery office | 13 (4.7) | 44 (9.9) |  |
|  | Primary care physician office | 17 (6.1) | 30 (6.8) |  |
|  | Plastic surgery office | 1 (0.4) | 2 (0.5) |  |
|  | General surgery office | 0 (0.0) | 1 (0.2) |  |
| Hand pain | Primary care physician office | 133 (47.7) | 261 (58.8) | 0.0032* |
|  | Urgent care | 93 (33.3) | 111 (25.0) |  |
|  | Emergency room | 51 (18.3) | 58 (13.1) |  |
|  | Orthopedic surgery office | 2 (0.7) | 13 (2.9) |  |
|  | Plastic surgery office | 0 (0.0) | 1 (0.2) |  |
| Nerve injury | Primary care physician office | 125 (45.1) | 189 (42.5) | <0.0001* |
|  | Emergency room | 74 (26.7) | 100 (22.5) |  |
|  | Orthopedic surgery office | 22 (7.9) | 102 (22.9) |  |
|  | Urgent care | 51 (18.4) | 42 (9.4) |  |
|  | Plastic surgery office | 4 (1.4) | 9 (2.0) |  |
|  | General surgery office | 1 (0.4) | 3 (0.7) |  |
| Cut finger off | Emergency room | 262 (93.9) | 408 (91.7) | 0.2798 |
|  | Primary care physician office | 9 (3.2) | 10 (2.2) |  |
|  | Urgent care | 5 (1.8) | 13 (2.9) |  |
|  | Orthopedic surgery office | 1 (0.4) | 8 (1.8) |  |
|  | Plastic surgery office | 2 (0.7) | 6 (1.3) |  |
| Deep laceration | Urgent care | 136 (48.9) | 175 (39.4) | 0.730 |
|  | Emergency room | 124 (44.6) | 228 (51.4) |  |
|  | Primary care physician office | 17 (6.1) | 30 (6.8) |  |
|  | Orthopedic surgery office | 1 (0.4) | 7 (1.6) |  |
|  | General surgery office | 0 (0.0) | 3 (0.7) |  |
|  | Plastic surgery office | 0 (0.0) | 1 (0.2) |  |
| Infection | Primary care physician office | 217 (78.1) | 312 (70.1) | <0.0001* |
|  | Orthopedic surgery office | 18 (6.5) | 74 (16.6) |  |
|  | Urgent care | 37 (13.3) | 32 (7.2) |  |
|  | Emergency room | 3 (1.1) | 16 (3.6) |  |
|  | General surgery office | 1 (0.4) | 8 (1.8) |  |
|  | Plastic surgery office | 2 (0.7) | 3 (0.7) |  |

*P-values were calculated using chi-square tests comparing the distribution of responses between respondents with and without a prior history of hand-related pathology for each scenario. This was an exploratory post hoc analysis and should be interpreted as hypothesis-generating.
