# Supplementary material for: How patients navigate specialist referral pathways in hand surgery: a cross-sectional survey study
Source: BMC Health Serv Res. 2026 May 30;26:1058. doi: 10.1186/s12913-026-14812-8 (PMC13439914; doi:10.1186/s12913-026-14812-8)
Supplement: Supplementary file 2 [file 12913_2026_14812_MOESM2_ESM.docx]

Survey Questions:

**Demographics**

1. What is your age? [Radio button, one choice]

- 18-29
- 30-39
- 40-49
- 50-59
- 60-69
- 70-79
- 80+

2. What is your gender? [Radio button, one choice]

- Female
- Male
- Other, prefer to self-identify

3. What is your race? [Checkbox, select all that apply]

- Native Hawaiian or other Pacific Islander
- Asian
- Black or African-American
- American Indian or Alaskan Native
- White
- Other

4. What is your ethnicity? [Radio button, one choice]

- Hispanic or Latino
- Not Hispanic or Latino
- Other
- More than one race

5. What is your highest level of education? [Radio button, one choice]

- 8th grade or less
- 9th to 12th grade, no diploma
- GED or high school graduate
- Some college, no degree
- Associates degree (2 year degree)
- Bachelors/college degree
- Some graduate work, no degree
- Graduate degree

6. What is your household income? [Radio button, one choice]

- Less than $9 999
- $10 000 to $24 999
- $25 000 to $49 999
- $50 000 to $74 999
- $75 000 to $99 999
- $100 000 to $149 999
- $150 000 or greater
- Prefer not to answer

7. Do you have health insurance?

[Yes/No]

If yes, check all that apply:

- Public
- Private
- Workers comp insurance
- Other

8. Do you have a primary care or regular doctor?

[Yes/No]

9. Have you ever seen a doctor for a hand-related issue?

- Yes [If yes, check box select all that apply: “It was an emergency” or “I chose to be treated for a non-urgent hand issue”]
- No
- Not sure

**Treating a New Hand-related Issue**

10. If I break my hand, my first call or visit would be to

- Emergency Room
- Urgent care
- My regular doctor’s office
- General Surgery department
- Orthopedic Surgery department
- Plastic Surgery department

11. If I have pain in my hand, my first call or visit would be to

- Emergency Room
- Urgent care
- My regular doctor’s office
- General Surgery department
- Orthopedic Surgery department
- Plastic Surgery department

12. If I injury a nerve in my hand, my first call or visit would be to

- Emergency Room
- Urgent care
- My regular doctor’s office
- General Surgery department
- Orthopedic Surgery department
- Plastic Surgery department

13. If I cut a finger off, my first call or visit would be to

- Emergency Room
- Urgent care
- My regular doctor’s office
- General Surgery department
- Orthopedic Surgery department
- Plastic Surgery department

14. If I have a deep cut on my hand, my first call or visit would be to

- Emergency Room
- Urgent care
- My regular doctor’s office
- General Surgery department
- Orthopedic Surgery department
- Plastic Surgery department

15. If I have an infection in my hand, my first call or visit would be to

- Emergency Room
- Urgent care
- My regular doctor’s office
- General Surgery department
- Orthopedic Surgery department
- Plastic Surgery department

**Choosing a Hand Surgeon**

16. If I need a hand surgeon, I want my primary care or regular doctor to refer me (send me to a doctor).
    a. [Likert scale, strongly agree, agree, N/P, disagree, strongly disagree] *
    b. *For REDCap, use a continuous bar scale of 5
17. If I need a hand surgeon, I would find one myself.

[Likert scale, strongly agree, agree, N/P, disagree, strongly disagree]

Dynamic form: If Likert scale is 4 or 5 - If I need a hand surgeon, I would find one by using:

[checkboxes, all that apply]

- PCP /regular doctor recommendation
- Ask my health insurance
- Search the internet
- Hospital Website
- Call the Hospital
- ask a friend

18. I would look for a hand surgeon in: [choose all that apply]

- Orthopedic Surgery
- Plastic and Reconstructive Surgery
- General Surgery
- I don’t know

19. My hand surgeon should have training in
    - Orthopedic surgery
    - Plastic and reconstructive surgery
    - Microsurgery
    - General surgery
20. The opinion of my friends or family would influence my decision of hand surgeon.

[Likert scale, strongly agree, agree, N/P, disagree, strongly disagree]

21. The opinion of my regular doctor would influence my decision of hand surgeon.

[Likert scale, strongly agree, agree, N/P, disagree, strongly disagree]

22. The doctor online ratings would influence my decision of hand surgeon.

[Likert scale, strongly agree, agree, N/P, disagree, strongly disagree]

23. My health insurance company would influence my decision of hand surgeon.

[Likert scale, strongly agree, agree, N/P, disagree, strongly disagree]

24. The location would influence my decision of hand surgeon.

[Likert scale, strongly agree, agree, N/P, disagree, strongly disagree]
